# In search of genome annotation consistency: solid gene clusters and how to use them

**DOI:** 10.1007/s13205-013-0152-2

**Published:** 2013-07-06

**Authors:** James J. Davis, Gary J. Olsen, Ross Overbeek, Veronika Vonstein, Fangfang Xia

**Affiliations:** 1 Institute for Genomic Biology, MC-195, University of Illinois at Urbana-Champaign, 1206 W. Gregory Dr., Urbana, IL 61801 USA; 2 Department of Microbiology, University of Illinois at Urbana-Champaign, 601 S. Goodwin Ave., Urbana, IL 61801 USA; 3 Fellowship for Interpretation of Genomes, 15W155 81st St., Burr Ridge, IL 60527 USA; 4 Mathematics and Computer Science, Argonne National Laboratory, 9700 S. Cass Ave., Argonne, IL 60439 USA

**Keywords:** Automatic annotation, Protein clusters

## Abstract

Maintaining consistency in genome annotations is important for supporting many computational tasks, particularly metabolic modeling. The SEED project has implemented a process that improves annotation consistencies across microbial genomes for proteins with conserved sequences and genomic context. In this research report, we describe this process and show how this effort has resulted in improvements to microbial genome annotations in the SEED. We also compare SEED annotation consistencies with other commonly used resources such as IMG (the Joint Genome Institute’s Integrated Microbial Genomes system), RefSeq (the National Center for Biotechnology Information’s Reference Sequence Database), Swiss-Prot (the annotated protein sequence database of the Swiss Institute of Bioinformatics, European Molecular Biology Laboratory and the European Bioinformatics Institute) and TrEMBL (Translated European Molecular Biology Laboratory nucleotide sequence data Library). Our analysis indicates that manual and computational efforts are paying off for the databases where consistency is a major goal.

## Introduction

The primary goal of the SEED Project is to produce accurate annotations for microbial genomes (Overbeek et al. 2005). Maintaining annotation consistency is a second major objective since it facilitates numerous computational tasks, notably the construction of metabolic models. In many contexts, it becomes important to determine, given two assigned functions, whether or not they refer to the same abstract function. To trivially illustrate what we mean by consistency (or lack of it), consider the following list of functions:

1. 50s ribosomal protein l34
2. LSU ribosomal protein L34p
3. Ribosomal protein L34
4. Ribosomal protein L34
5. Ribosomal protein L34 RpmH
6. RpmH
7. rpmH gene product

These are all alternative names of the same function, and they all occur within the public repositories. While heuristic tools can be developed to allow recognition of variants, it is less cumbersome to seek accurate and identical representations of each function. Thus, rather than attempting to computationally determine that these are all equivalent, we have attempted to unify these variants within the SEED Project. For instance, in this case we use only the second annotation from the list above.

To be clear, we wish to approach consistency in annotations to support automated construction of metabolic (and more general) models based upon the annotated functions of the genes. We are not concerned with a global standard in nomenclature since sets of terms that accurately and consistently reflect the functions of proteins can be automatically mapped to one another through the associated protein sequences. Secondly, we are not intending to reflect chromosomal location or expression in the function of the protein, but rather the function that it would perform if it was expressed in a cell, again, with a goal toward modeling and metabolic engineering.

Given the goal of representing the activity (or other function) of gene products, the most obvious first step in building and maintaining annotation consistency between genomes is to apply a standard (within the given genome database) nomenclature among proteins with identical primary sequences. In addition, there are many instances where conserved sequence similarity and genomic context offer abundant evidence for annotating a given gene. This report describes simple tools that we have constructed for estimating conserved gene clusters within an operational taxonomic unit (OTU), guiding highly reliable projections of function within the OTU, constructing sets of proteins believed to implement identical functions, and using these sets to estimate the consistency of a set of annotations.

## Description of the algorithm

There are many instances where protein-encoding genes with highly conserved amino acid sequence and genomic context can be safely annotated based on the annotation of genomes that have already been sequenced. The following steps describe how we chose our sets of gene clusters with conserved genomic context.

*Step 1. The microbial genomes in the SEED database are separated into Operational Taxonomic Units (OTUs).* We define an OTU as a set of genomes that are ≥97 % identical in their 16S rDNA genes (e.g. Schloss and Handelsman 2005). At the time of this study (February, 2013), there were 1,386 OTUs represented in the SEED. OTUs containing less than five genomes were omitted from subsequent steps, and this resulted in a total of 100 OTUs, containing 4,117 microbial genomes analyzed in this study.

*Step 2. A focus organism representing an OTU is chosen.*

*Step 3. A set of organisms, moderately related to the focus organism, is chosen*. It is necessary to find a set of organisms that are related to the focus organism to determine if the context of each gene is conserved. In this case, closely related strains are avoided because their genomic context is too strongly conserved, but more distantly related organisms are less conserved and are thus more useful for determining if a given gene has a conserved context. Our set of related organisms is defined as those that are between 50 and 90 % amino acid identity from the focus organism and >90 % identical to one another. Percent identity is determined from a concatenated alignment of aminoacyl-tRNA synthetase proteins (AARS). This alignment includes all of the bacterial and archaeal genomes in the SEED database, and contains all of the AARS proteins except for the asparaginyl-, glutaminyl-, glycyl- and lysyl-tRNA synthetases, which were excluded because they are absent or nonhomologous in many taxa (Woese et al. 2000). From this set of related organisms, a representative set that has less than 90 % protein identity from each other is chosen. It must be noted that there has been extensive horizontal gene transfer among the AARS proteins and that their concatenated alignment does not necessarily provide an accurate phylogeny outside of a given OTU (Woese et al. 2000). We use them in this context because they are among the best-annotated genes in the SEED and their concatenated alignment provides a suitable frame of reference, although almost any highly conserved protein or rRNA alignment with adequate taxonomic representation would suffice.

*Step 4. Gene clusters in the genome of the focus organism are chosen for analysis.* In order to determine the regions of conserved contiguity, we search for gene sets in which contiguity is maintained in the focus genome and throughout the set of moderately related organisms. This search is performed by taking two genes occurring close to one another in the genome of the focus organism, and determining whether the same pair of genes also occurs in close proximity throughout the genomes of the moderately related set. If there is substantial preservation of contiguity, we treat the two genes in the reference genome as part of a single cluster and these binary connections are used to form larger clusters (using single-linkage clustering). We define substantial preservation of contiguity as follows: for each pair of genes in the reference genome that is separated by less than five intervening genes, we look for bidirectional best hits (BBH) in each of the moderately related genomes. We restrict the usual notion of BBHs (e.g. Overbeek et al. 1999) to genes that have protein products that are reciprocal best hits, similar over 80 % of each protein, and at least 50 % identical over the region of similarity using BLASTP (Altschul et al. 1997). Then in each moderately related genome with a pair of BBH proteins, we look for conserved location of the corresponding gene pair using the same parameters as above (they must have no more than five intervening genes). For a given pair of genes in the focus organism to be considered as a cluster, or as members of a larger cluster, the pair must have a conserved location in 40 % of the genomes of the moderately related set of organisms.

*Step 5. Gene clusters are populated.* Once we have generated estimates of the gene clusters in the genome of the focus organism, we project these potential clusters (again, very conservatively) to all of the genomes within the same OTU. Here the same parameters from step 4 are used, and we also require that conserved contiguity be detected in at least five genomes or in 20 % of the genomes of the OTU, whichever is larger. We call the set of clustered genes passing all of the above criteria and projecting throughout the OTU a “Solid Cluster”. We tabulate these solid clusters in the form of tables in which each row represents a single genome from the OTU, and each column contains one gene in the reference genome and the corresponding BBHs in the other genomes from the OTU. Each column in each of these tables constitutes a “Solid Set” which is believed to be composed of isofunctional homologs.

There are a number of parameters in this approach relating to the definition of “the generation of OTUs”, “closeness of gene pairs”, “BBHs”, and “conserved contiguity”. In this report, we do not explore the optimization of each individual parameter. In all cases, we chose relatively conservative values because they are ultimately linked to the automated propagation of gene annotations in the SEED (see below). We certainly acknowledge that loosening these parameters can lead to larger clusters covering more of the genes within the reference genome, but that this may also increase projection errors.

*Step 6. The Solid Clusters are retained, and steps 1*–*4 are repeated for other focal genomes from different OTUs.*

## Using clusters to evaluate the consistency of annotations

We propose that a manual annotation assigned to an individual protein-encoding gene occurring in a solid set should propagate to all of the protein sequences occurring in the solid set. We have implemented this within the SEED Project in an attempt to project the relatively expensive manual annotations. Thus, a single manual assignment done in a genus in which hundreds of genomes exist (a situation that is rapidly beginning to happen) may induce hundreds of annotation updates.

The existence of a collection of solid sets makes it possible to easily define a number of metrics to measure the consistency of annotations. For a number of annotation efforts, we have chosen to measure two values:

1. Given two genes encoding identical proteins, what is the frequency of identical assigned functions?
2. Given two genes encoding two proteins from the same solid set, what is the frequency of identical assigned functions?

We have computed Solid Clusters for 100 distinct OTUs that were present in the PubSEED. This led to the formation of 73,093 distinct solid sets, with each set believed to contain proteins implementing a common function. Table 1 shows these values for several collections of annotated proteins, which were downloaded in February of 2013 (Lima et al. 2009; Markowitz et al. 2012; O’Donovan et al. 2002; Overbeek et al. 2005; Pruitt et al. 2007). The collections analyzed are IMG (ftp://downloads1.jgi-psf.org/pub/IMG/img_core_v400.tar), RefSeq (http://ftp.ncbi.nih.gov/blast/db/FASTA/nr.gz), the SEED (ftp://ftp.theseed.org/misc/annotation/seed.fa), Swiss-Prot (ftp://ftp.uniprot.org/pub/databases/uniprot/current_release/knowledgebase/complete/uniprot_sprot.fasta.gz) and TrEMBL (ftp://ftp.uniprot.org/pub/databases/uniprot/current_release/knowledgebase/complete/uniprot_trembl.fasta.gz). In each case, we have tabulated the number of sequences from the publicly distributed collection that have identical protein sequences occurring within solid sets, as well as the two metrics. The data in the table clearly indicate that the efforts expended in Swiss-Prot and the SEED Projects have led to significant advances in annotation consistency.

**Table 1:** Measured inconsistencies in annotations

Source of annotations	Fraction of proteins in the database occurring in solid sets	Unique protein sequences occurring in solid sets	Metric 1 (frequency of inconsistency given identical proteins)	Metric 2 (frequency of inconsistency, among members of a solid set)
IMG	0.048	436,872	0.640	0.697
RefSeq	0.022	459,433	0.564	0.625
SEED	0.135	538,181	0.023	0.037
Swiss-Prot	0.206	67,972	0.039	0.039
TrEMBL	0.085	419,239	0.341	0.396

Overall, the fraction of proteins in each database that are currently represented by solid sets is low, ranging from 0.048 in IMG to 0.206 in Swiss-Prot. This range differs because of the presence of eukaryotic proteins (which are not currently analyzed), the density of genome sequences for a given OTU, and the parameters of the algorithm. The percentage of individual genomes encoding proteins covered by solid sets ranges from 0 to 56 %, with the genome of *Buchnera aphidicola* strain APS having the highest coverage. In general, for OTUs that are rich in genomic data, we observe more proteins encoded by the genome occurring in solid sets. For instance, in *Escherichia coli* K-12 45 % of the proteins encoded by the genome are covered by solid sets. As sequence data continue to accumulate, solid sets will cover a larger fraction of the genomes in more diverse OTUs.

It is important to note that consistency is not the sole goal of most annotation projects. Accuracy of the annotation is clearly more important (Chen et al. 2013). For instance, the eight ribosomal proteins mentioned in the introduction, while inconsistent, could all be viewed as being accurate. Furthermore, they could all be viewed as being consistent in the eyes of an expert annotator. In this report, we have not attempted to assess the absolute accuracy in the databases. Instead we have focused on consistency, primarily to support the automated steps necessary in model building (i.e., that the same string of characters in the annotation is assigned to proteins implementing the same abstract function).

The topic of consistency is closely related to the use of a controlled vocabulary. We have chosen to use the SEED functional roles. They have been adopted by the Model SEED metabolic modeling framework which has constructed thousands of metabolic models using the SEED’s controlled vocabulary (Henry et al. 2010), and more recently by the US Department of Energy’s Kbase project (www.kbase.us). These resources make it possible to automatically reconstruct the metabolic network (or a good approximation of it) from just the list of functional roles associated with the genes in a genome, if (and only if) there exists a consistently used controlled vocabulary and one has a table associating reactions with the functional roles corresponding to the enzymes that catalyze the reactions. The Model SEED and Kbase projects include a precise correspondence between a subset of the SEED functional roles and the reactions these functional roles enable.

## Summary

In this report, we have described a simple technology for generating sets of proteins from a single OTU that are believed to implement identical functions. What distinguishes this effort from other well-known projects to construct protein families is that the Solid Clusters are populated very conservatively, leading to sets that only cover proteins encoded by genomes from a single OTU and are of high reliability. Furthermore, since the generation of solid clusters is fully automated, it provides a complementary approach to traditional methods of genome annotation that use hierarchical annotation structures such as SEED Subsystems, GO terms and COGs (Ashburner et al. 2000; Overbeek et al. 2005; Tatusov et al. 2003).

We have made the Solid Clusters, along with the generated sets of proteins available on the PubSEED web site (ftp://ftp.theseed.org/misc/annotation/). We used these sets to evaluate the consistency of existing sets of annotations from a number of sources. We will periodically update the relevant datasets, allowing any group to evaluate their annotations using this metric, and the evaluation of commonly used sources of annotations.
